# When stress meets the feed: algorithmic curation, digital stress relief, and academic amotivation in the attention economy

**DOI:** 10.3389/fpsyg.2026.1787564

**Published:** 2026-04-16

**Authors:** Jing Jin, XiaCheng Song, Lu Sun, Xiqiong Yi, Quanyue Zheng

**Affiliations:** 1 Guangdong University of Science and Technology, Dongguan, China; 2 Xi'an Fanyi University, Xi'An, China; 3 Dongguan Polytechnic, Dongguan, China; 4 Shinawatra University, Pathum Thani, Thailand

**Keywords:** academic amotivation, algorithmic curation, immersive escapism, pressure uncertainty, self-regulatory fatigue

## Abstract

Algorithmically curated feeds have become a default layer of everyday life, offering rapid affect regulation under academic pressure while also reshaping attention and self-regulation. Moving beyond the generic “screen time” debate, this study examines a socio-technical pathway in which perceived pressure uncertainty is associated with academic amotivation through immersive escapism and self-regulatory fatigue. We surveyed 518 university students in China and measured pressure uncertainty, immersive escapism, self-regulatory fatigue, and academic amotivation. Using confirmatory factor analysis and latent structural equation modeling with ordinal indicators, while controlling demographics and entertainment-focused scrolling time, we found that pressure uncertainty was positively associated with immersive escapism. Immersive escapism, in turn, was positively associated with self-regulatory fatigue, which was associated with higher academic amotivation. Competing path models were more consistent with the hypothesized ordering than with alternative specifications. These findings suggest that platform-shaped digital relief may be linked to a depletion-oriented coping loop with educational consequences, pointing to intervention leverage points that are more actionable than broad calls to simply “use less.” We discuss implications for higher education (digital wellbeing support, self-regulation scaffolding, and algorithm-related digital literacy) and for platform design and accountability (greater user control, transparency, and pacing mechanisms that may interrupt maladaptive loops).

## 1 Introduction

Academic pressures are becoming volatile, competitive, and unpredictable to university students, who have to navigate under these conditions (Koppenborg et al., 2024; Li et al., 2025; Pérez-Jorge et al., 2025). Here, digital media can be seen as a relief that can be accessed instantly, a resource that students can turn to when they are in a strained situation without the necessity of time, money, or social organization (Chen J. et al., 2025; Ghanayem et al., 2024; Shiraly et al., 2024). However, not every digital relief is psychologically equal. Most of the most successful platforms are designed on the basis of algorithmically curated feeds that constantly provide new content and reduce natural stopping points (de Segovia Vicente et al., 2024; Metzler and Garcia, 2024; Taylor and Chen, 2024). In the event that stress relief is sought within such settings, the question at hand is no longer whether or not students are using their phones, but how the engagement that is being shaped by an algorithm can change coping in a manner that incurs downstream costs to self-regulation and academic motivation (Khalaf et al., 2025; Obeng et al., 2025; Shiraly et al., 2024).

Although the literature on mobile media and its effects on student wellbeing is rapidly growing, much of the evidence remains focused on broad measures of exposure (frequency or time spent) or high-level measures (problematic use) (Chen et al., 2024; Li et al., 2023; Timpano and Beard, 2020). The methods may hide the process through which the experiences associated with pressure lead to continued involvement and, ultimately, academic performance. Specifically, there are three gaps in understanding that are conceptual (Chen J. et al., 2025; Pérez-Jorge et al., 2025). To begin with, the platform-specific aspect that is most likely to be relevant, which is algorithm-driven immersion that renders disengagement a strenuous process, is commonly discussed as a backdrop as opposed to a component of the process (Chen Y. et al., 2025; Shahzad et al., 2025; Shiraly et al., 2024). Second, escapist coping is widely claimed to be a motive but rarely theorized as a step in a sequence of ordered responses between stressors and resource depletion. Third, correlates often focus on downstream effects of academic motivation, and it remains unclear how stress, immersion, coping, and fatigue interact to form a coherent system (Cao and Meng, 2022; Orazi et al., 2023). To fill these gaps, a mechanism-based explanation is needed, which defines the point at which, within the chain of stress to academic functioning, algorithm-shaped immersion has its effect (Gabbiadini et al., 2021; Soraci et al., 2025).

The current research contributes to this mechanism-based agenda by examining a serial process model in which pressure uncertainty (PU) is associated with academic amotivation (AA) through immersive escapism (IE) and self-regulatory fatigue (SRF). Conceptually, PU refers to students' perceived experience of academic and future-related pressure as unstable, difficult to anticipate, and difficult to manage in a psychologically orderly way. In digitally saturated environments, this condition may also heighten sensitivity to the perceived unpredictability of algorithmically curated content. In the present study, this construct is not intended to capture algorithmic awareness, information overload, or generalized perceptions of platform control; rather, it refers to a pressure-related condition under which students may be more likely to seek low-friction digital relief in recommendation-driven environments. IE refers to a coping-oriented mode of digital immersion in which students turn to continuously refreshed, algorithmically curated content for short-term relief or psychological distance from stressors, whereas SRF refers to the depletion of self-regulatory resources that may follow repeated or prolonged reliance on such coping. AA, in turn, reflects reduced meaning, initiative, and motivation toward academic pursuits (Nweke et al., 2024; Qiang et al., 2024; Yuhuan et al., 2022). To improve interpretability, we examine the measurement structure of these constructs, test whether the measures function similarly across gender, and compare the hypothesized serial pathway with theoretically plausible competing specifications, including parallel and reverse-order models (Gonzalez et al., 2025; Li et al., 2023). This approach moves beyond descriptive associations by evaluating whether the observed data are more consistent with an ordered mechanism than with a loosely connected set of predictors.

Under the influence of these purposes, we answer the following research questions:

RQ1. How do self-reported digital stress-relief tools (e.g., short-video platforms, music, games, social media, and AI chat/companion apps) of students in this sample distribute?

RQ2. Do immersive escapism (IE) and pressure uncertainty (PU) relate when considering key demographics and entertainment-focused scrolling time?

RQ3. Does the serial pathway PU → IE → SRF explain the association between PU and academic amotivation (AA), and does PU retain a direct association with AA (i.e., partial mediation)?

RQ4. Does the proposed serial model offer a superior explanation of the data as compared to the theoretically plausible competing specifications (parallel and reversed-order models)?

## 2 Methods

### 2.1 Study design and setting

This study used a cross-sectional design and an anonymous, self-administered online questionnaire (von Elm et al., 2007). Data were collected over a 1-week period from students enrolled at a private undergraduate university in China. The survey was administered through an online survey platform and distributed within the university through routine institutional communication channels. Participants were informed that participation was voluntary, that no incentives were provided, and that they could discontinue at any point prior to submission.

### 2.2 Respondents and recruitment

The survey invitation and access link were distributed within the university by teachers, counselors, and student organization representatives through routine student communication channels. Eligibility was limited to currently enrolled students of the focal institution who provided electronic informed consent. Participation was voluntary and unpaid. A total of 518 valid completed responses were retained for analysis (*N* = 518).

### 2.3 Measures

All focal constructs were assessed using brief multi-item measures included in the questionnaire. Items were rated on 5-point Likert-type response scales, with higher scores indicating higher levels of the construct; no items were reverse-coded. The measurement strategy was theory-driven: item sets were developed or adapted to reflect the conceptual structure of the proposed model rather than to reproduce any single pre-existing scale in full. Pressure uncertainty (PU) captured students' perceived experience of academic and future-related pressure as unstable, difficult to anticipate, and difficult to manage. Immersive escapism (IE) captured coping-oriented immersion in mobile content environments, especially patterns of stress relief through difficult-to-disengage, continuously refreshed, algorithmically curated content. Self-regulatory fatigue (SRF) assessed perceived depletion of self-control, persistence, and regulatory capacity. Academic amotivation (AA) assessed weakened meaning, initiative, and motivation toward academic pursuits. Given the brevity of the instrument and the use of ordinal indicators, composite scores were used only for descriptive purposes, whereas the main inferential analyses were conducted at the latent-variable level (DiStefano et al., 2009).

### 2.4 Ethics and protection of the participants

The institutional ethics committee of the authors reviewed and approved the study protocol. Electronic informed consent was obtained prior to participation. No direct identifiable data was gathered; the answers were anonymous, were stored safely and were only accessed as research data. The participation was voluntary and the respondents were told that they would not be penalized and could leave the survey any time they wanted.

### 2.5 Data preparation and screening

The analysis of data was preceded by screening data on completeness and basic plausibility. The final dataset was based on completed survey submissions (*N* = 518) as the major inclusion criterion; any occasional item-level missingness was handled as described below. Variables were processed according to their level of measurement: individual items were treated as ordered categorical indicators in confirmatory factor analysis (CFA) and SEM, whereas composite scale scores were used only for descriptive summaries. Missingness was handled in a model-consistent fashion (see Section 2.6) and no item-level imputation was performed.

### 2.6 Statistical analysis

Analyses were conducted in R. Descriptive statistics and bivariate correlations were computed, and internal consistency was assessed using Cronbach's alpha and McDonald's omega. The measurement model was examined using a four-factor confirmatory factor analysis (CFA) with ordered categorical indicators and a robust estimator appropriate for ordinal data (WLSMV) (Muthén, 1984; Rosseel, 2012). Model fit was evaluated using standard indices (CFI, TLI, RMSEA, and SRMR) and standardized loadings (Hu and Bentler, 1999; Kline, 2016). Convergent and discriminant validity were evaluated using composite reliability (CR), average variance extracted (AVE), heterotrait–monotrait ratios (HTMT), and the Fornell–Larcker criterion. Measurement invariance across gender was assessed via multi-group CFA in a stepwise sequence (configural, metric, and scalar), with changes in approximate fit indices (e.g., ΔCFI/ΔRMSEA) used to evaluate invariance (Chen, 2007; Putnick and Bornstein, 2016). The hypothesized serial mediation model was then tested using a full latent structural equation model estimated with WLSMV, specifying paths from Pressure uncertainty (PU) to immersive escapism (IE) to self-regulatory fatigue (SRF) to academic amotivation (AA), while also including a direct path from PU to AA and covariates (gender, grade level, and daily entertainment-scrolling time). Gender was dummy-coded (0 = female, 1 = male), and grade level and scrolling time were coded ordinally to reflect increasing levels and treated as ordered continuous covariates. Indirect effects were evaluated using model-implied estimates, and uncertainty was summarized using robust standard errors and 95% confidence intervals; bootstrap approaches are also commonly used for mediation inference (Hayes, 2013; Preacher and Hayes, 2008). Competing specifications (serial, parallel, reverse-order) were compared using approximate fit indices (scaled χ^2^, CFI, RMSEA, and SRMR); because information criteria (AIC/BIC) are not available for WLSMV and incremental fit indices can behave unexpectedly in highly constrained or near-saturated contexts, comparisons relied primarily on these fit indices (Akaike, 1974; Schwarz, 1978). Given the self-report, single-survey design, common method bias cannot be ruled out entirely. However, this risk was reduced through anonymous and voluntary responding, the use of separate multi-item indicators for the focal constructs, and the evaluation of discriminant validity within the measurement model.

### 2.7 Transparency and open science

The study was not pre-registered (Nosek et al., 2015). Data, analysis scripts and study materials will be posted in an open repository and made publicly available when the study is published, in line with ethical and institutional requirements and without jeopardizing the confidentiality of participants.

## 3 Results

### 3.1 Sample characteristics and descriptive overview

**Figure 1:**
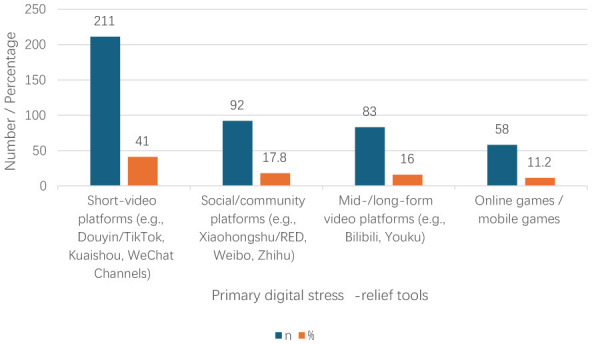
Distribution of primary digital stress-relief tools (*N* = 518). Bars indicate the number of participants (*n*) and the corresponding percentage (%) for each category. Percentages are computed based on non-missing responses and may not sum to 100% due to rounding.

As shown in Table 1, the analytic sample comprised 518 participants, most of whom reported an arts and design background (*n* = 315, 61%), followed by humanities and social sciences (*n* = 117, 23%) and STEM/health-related majors (*n* = 86, 17%). The sample included 328 females (63%) and 190 males (37%). In terms of grade level, 275 participants were freshmen/sophomores (53%), 164 were juniors/seniors (32%), and 79 were master's/doctoral students (15%). Regarding daily time spent on entertainment scrolling, 91 participants reported ≤ 2 h (18%), 155 reported 2–4 h (30%), 167 reported 4–6 h (32%), and 105 reported ≥6 h (20%). For the primary digital stress-relief tool, short-video platforms were most frequently selected (*n* = 211, 41%), followed by social/text-based communities (*n* = 92, 18%), long-form video platforms (*n* = 83, 16%), online games/mobile games (*n* = 58, 11%), online novels/comics (*n* = 52, 10%), and AI chat/companion apps (*n* = 22, 4%). Figure 1 presents the distribution of the primary digital stress-relief tools reported by participants.

**Table 1:** Participant characteristics (*N* = 518).

Variable	Category	*n* (%)
Major background	Arts and design (e.g., visual communication, product design, digital media)	315 (60.8%)
	Humanities and social sciences (e.g., arts/humanities, business/law, education/psychology)	117 (22.6%)
	STEM and health-related majors (e.g., computer science, engineering, mathematics, medicine)	86 (16.6%)
Gender	Male	190 (36.7%)
	Female	328 (63.3%)
Grade level	Freshman/sophomore	275 (53.1%)
	Junior/senior	164 (31.7%)
	Master's/doctoral	79 (15.3%)
Daily time spent on entertainment scrolling	≤ 2 h	91 (17.6%)
	2–4 h	155 (29.9%)
	4–6 h	167 (32.2%)
	≥6 h	105 (20.3%)
Primary digital stress-relief tool	Short-video platforms (e.g., Douyin/TikTok, Kuaishou, WeChat Channels)	211 (40.7%)
	Social/text-based communities (e.g., Xiaohongshu/RED, Weibo, Zhihu)	92 (17.8%)
	Long-form video platforms (e.g., Bilibili, Youku)	83 (16.0%)
	Online games/mobile games	58 (11.2%)
	Online novels/comics	52 (10.0%)
	AI chat/companion apps (e.g., Wenxin Yiyan, Kimi, Doubao, Xingye, Zhumeidao)	22 (4.2%)

Values are reported as *n* (%). Percentages are calculated within each variable based on available responses and are rounded to one decimal place; totals may not sum to 100% due to rounding.

The most commonly reported primary tool of digital stress relief was a short-video platform (*n* = 211, 41%), followed by social/text-based communities (*n* = 92, 18%), long-form video platforms (*n* = 83, 16%), online games/mobile games (*n* = 58, 11%), online novels/comics (*n* = 52, 10%), and AI chat/companion apps (*n* = 22, 4%).

### 3.2 Measurement model: CFA and reliability/validity evidence

Table 2 summarizes descriptive statistics and internal consistency indices for the study constructs (*N* = 518). Mean levels were comparable across constructs (*M* = 2.842–3.121; SD = 0.829–0.864), with immersive escapism (IE) showing the highest mean (*M* = 3.121, SD = 0.829) and academic amotivation (AA) the lowest (*M* = 2.842, SD = 0.864). Internal consistency was modest overall: Cronbach's α ranged from 0.583 (AA) to 0.720 (IE), and McDonald's ωt (polychoric) ranged from 0.636 (AA) to 0.762 (IE), with intermediate values for pressure uncertainty (PU; α = 0.617, ωt = 0.661) and self-regulatory fatigue (SRF; α = 0.699, ωt = 0.741).

**Table 2:** Descriptive statistics and internal consistency of study constructs (*N* = 518).

Construct	Items (k)	*M*	SD	Cronbach's α	McDonald's ωt (polychoric)
PU	4	2.843	0.831	0.617	0.661
IE	5	3.121	0.829	0.720	0.762
SRF	4	2.939	0.858	0.699	0.741
AA	3	2.842	0.864	0.583	0.636

*M* and SD are computed from participants' mean scale scores. Cronbach's α is reported as a reference index; McDonald's ωt is estimated based on polychoric correlations (one-factor solution) for ordinal indicators. PU, pressure uncertainty; IE, immersive escapism; SRF, self-regulatory fatigue; AA, academic amotivation. Values are rounded to three decimals.

Table 3 presents standardized factor loadings from the CFA measurement model estimated with WLSMV using ordered categorical indicators (*N* = 518). Loadings for pressure uncertainty (PU) ranged from 0.478 to 0.675 across Q1, Q2, Q3, and Q17; loadings for immersive escapism (IE) ranged from 0.543 to 0.697 across Q5, Q7, Q9, Q10, and Q11; loadings for self-regulatory fatigue (SRF) ranged from 0.550 to 0.729 across Q13–Q16; and loadings for academic amotivation (AA) ranged from 0.516 to 0.755 across Q18–Q20, with all loadings statistically significant (*p* < 0.001). Overall model fit was good (CFI = 0.986, TLI = 0.983, RMSEA = 0.049, SRMR = 0.047), supporting the adequacy of the four-factor measurement model for subsequent analyses.

**Table 3:** Standardized factor loadings for the CFA measurement model (WLSMV; *N* = 518).

Factor	Item	Standardized loading (λ)	*p*
Pressure uncertainty (PU)	Q1	0.488	<0.001
Pressure uncertainty (PU)	Q2	0.478	<0.001
Pressure uncertainty (PU)	Q3	0.630	<0.001
Pressure uncertainty (PU)	Q17	0.675	<0.001
Immersive escapism (IE)	Q5	0.613	<0.001
Immersive escapism (IE)	Q7	0.543	<0.001
Immersive escapism (IE)	Q9	0.697	<0.001
Immersive escapism (IE)	Q10	0.687	<0.001
Immersive escapism (IE)	Q11	0.594	<0.001
Self-regulatory fatigue (SRF)	Q13	0.550	<0.001
Self-regulatory fatigue (SRF)	Q14	0.700	<0.001
Self-regulatory fatigue (SRF)	Q15	0.729	<0.001
Self-regulatory fatigue (SRF)	Q16	0.610	<0.001
Academic amotivation (AA)	Q18	0.547	<0.001
Academic amotivation (AA)	Q19	0.516	<0.001
Academic amotivation (AA)	Q20	0.755	<0.001

CFA, confirmatory factor analysis. Standardized factor loadings (λ) are from the standardized solution (std.all). The model was estimated using WLSMV with ordered categorical indicators. Model fit: CFI = 0.986, TLI = 0.983, RMSEA = 0.049, SRMR = 0.047. Values are rounded to three decimals; *p* values < 0.001 are reported as “ < 0.001.”

Table 4 reports discriminant validity evidence using heterotrait–monotrait ratios (HTMT) among the four constructs (PU, IE, SRF, AA). The HTMT values ranged from 0.553 to 0.856, with the largest ratio observed between immersive escapism (IE) and self-regulatory fatigue (SRF; HTMT = 0.856). The remaining HTMT values were 0.715 (PU–IE), 0.729 (PU–SRF), 0.688 (PU–AA), 0.553 (IE–AA), and 0.625 (SRF–AA), indicating that the strongest overlap was concentrated in the IE–SRF pairing, whereas the other construct pairs showed comparatively lower HTMT values.

**Table 4:** Discriminant validity: Heterotrait–Monotrait ratios (HTMT) among study constructs (*N* = 518).

Construct	PU	IE	SRF	AA
PU	—	0.715	0.729	0.688
IE	0.715	—	0.856	0.553
SRF	0.729	0.856	—	0.625
AA	0.688	0.553	0.625	—

HTMT, heterotrait–monotrait ratio of correlations. Values closer to 1.00 indicate weaker discriminant validity; commonly used heuristic cutoffs include 0.85 (more conservative) and 0.90 (less conservative). PU, pressure uncertainty; IE, immersive escapism; SRF, self-regulatory fatigue; AA, academic amotivation. Values are rounded to three decimals.

### 3.3 Measurement invariance across gender

Table 5 summarizes multi-group CFA measurement invariance across gender for the CFA model estimated with WLSMV. The configural model showed CFI = 0.921 and RMSEA = 0.076, supporting an acceptable baseline structure across groups. Imposing equality constraints on factor loadings (metric invariance) yielded CFI = 0.935 and RMSEA = 0.067 (ΔCFI = 0.014; ΔRMSEA = −0.009 relative to the configural model), and further constraining both loadings and thresholds (scalar invariance) produced CFI = 0.926 and RMSEA = 0.066 (ΔCFI = −0.009; ΔRMSEA = 0.000 relative to the metric model). Together, these results indicate that model fit remained broadly comparable across increasingly restrictive invariance specifications as reported in Table 5.

**Table 5:** Measurement invariance across gender for the CFA model (WLSMV; *N* = 518).

Model	CFI	RMSEA	ΔCFI	ΔRMSEA
Configural	0.921	0.076	—	—
Metric (loadings)	0.935	0.067	0.014	−0.009
Scalar (loadings + thresholds)	0.926	0.066	−0.009	0.000

Multi-group CFA measurement invariance was tested across gender using WLSMV with ordered categorical indicators. Metric invariance constrains factor loadings to be equal across groups; scalar invariance constrains factor loadings and item thresholds to be equal across groups. Changes in model fit (ΔCFI, ΔRMSEA) are computed relative to the preceding model. Values are rounded to three decimals.

### 3.4 Structural model: direct effects

**Figure 2:**
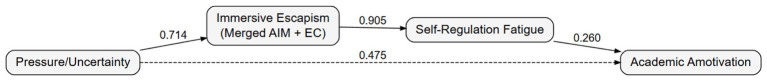
Estimated serial mediation model with standardized path coefficients. Values on arrows are standardized path coefficients (β) from the fitted serial mediation model (PU → IE → SRF → AA) with a direct path from PU to AA (dashed line). Gender, grade level, and daily entertainment-scrolling time were included as covariates in all structural equations but are omitted for clarity. PU, pressure uncertainty; IE, immersive escapism; SRF, self-regulatory fatigue; AA, academic amotivation.

Figure 2 depicts the hypothesized serial mediation model with standardized path coefficients. Pressure uncertainty (PU) was positively associated with immersive escapism (IE; β = 0.714), and IE was strongly associated with self-regulatory fatigue (SRF; β = 0.905). SRF, in turn, was positively related to academic amotivation (AA; β = 0.260), and a direct path from PU to AA remained positive (β = 0.475), consistent with the model structure shown in Figure 2. Gender, grade level, and daily entertainment-scrolling time were included as covariates in all structural equations but are not displayed in the figure for clarity.

Table 6 presents the full latent SEM estimates (WLSMV) corresponding to Figure 2, including covariates. The core structural paths were all statistically significant: PU predicted IE (β = 0.714, *p* < 0.001), IE predicted SRF (β = 0.905, *p* < 0.001), SRF predicted AA (β = 0.260, *p* = 0.002), and PU also directly predicted AA (β = 0.475, *p* < 0.001). Among covariates, gender (0 = female, 1 = male) was associated with IE (β = −0.171, *p* < 0.001) and SRF (β = 0.113, *p* = 0.012), grade level was associated with SRF (β = −0.092, *p* = 0.017), and daily entertainment-scrolling time was associated with IE (β = 0.177, *p* < 0.001) and AA (β = 0.137, *p* = 0.007), whereas the remaining covariate effects reported in Table 6 were not statistically significant.

**Table 6:** Full latent structural equation model results with covariates (WLSMV; *N* = 518).

Dependent variable	Predictor	β (standardized)	*B*	SE	*p*
Immersive escapism (IE)	Pressure uncertainty (PU)	0.714	1.095	0.119	<0.001
Immersive escapism (IE)	Gender (0 = female, 1 = male)	−0.171	−0.544	0.162	<0.001
Immersive escapism (IE)	Grade level	0.039	0.082	0.096	0.396
Immersive escapism (IE)	Daily entertainment-scrolling time	0.177	0.271	0.074	<0.001
Self-regulatory fatigue (SRF)	Immersive escapism (IE)	0.905	1.341	0.178	<0.001
Self-regulatory fatigue (SRF)	Gender (0 = female, 1 = male)	0.113	0.531	0.211	0.012
Self-regulatory fatigue (SRF)	Grade level	−0.092	−0.283	0.119	0.017
Self-regulatory fatigue (SRF)	Daily entertainment-scrolling time	0.024	0.055	0.095	0.567
Academic amotivation (AA)	Self-regulatory fatigue (SRF)	0.260	0.160	0.050	0.002
Academic amotivation (AA)	Pressure uncertainty (PU)	0.475	0.663	0.126	<0.001
Academic amotivation (AA)	Gender (0 = female, 1 = male)	0.036	0.103	0.145	0.477
Academic amotivation (AA)	Grade level	0.065	0.123	0.088	0.160
Academic amotivation (AA)	Daily entertainment-scrolling time	0.137	0.191	0.071	0.007

Results are from the full latent SEM estimated using WLSMV with ordered categorical indicators. β denotes standardized coefficients (std.all). Gender was dummy-coded (0 = female, 1 = male). Grade level and daily entertainment-scrolling time were treated as continuous covariates. Values are rounded to three decimals; *p* values <0.001 are reported as “ <0.001.”

### 3.5 Mediation and robustness checks

Table 7 reports the indirect and total effects from the hypothesized serial mediation model estimated with WLSMV (*N* = 518). The serial indirect effect (ind_chain), defined as pressure uncertainty (PU) → immersive escapism (IE) → self-regulatory fatigue (SRF) → academic amotivation (AA), was statistically significant (*B* = 0.234, SE = 0.075, *p* = 0.002), with a 95% confidence interval that did not include zero [CI (0.086, 0.382)] and a standardized estimate of β = 0.168. The total effect was also significant (*B* = 0.898, SE = 0.100, *p* < 0.001), with a 95% confidence interval of [0.702, 1.093] and a standardized estimate of β = 0.643, indicating that PU was positively associated with AA when accounting for the modeled indirect pathway.

**Table 7:** Indirect and total effects in the hypothesized serial mediation model (WLSMV; *N* = 518).

Effect	Estimate (*B*)	SE	*p*	95% CI (lower)	95% CI (upper)	Standardized estimate (β)
Ind_chain	0.234	0.075	0.002	0.086	0.382	0.168
Total	0.898	0.100	<0.001	0.702	1.093	0.643

The indirect effect (ind_chain) represents the serial pathway pressure uncertainty (PU) → immersive escapism (IE) → self-regulatory fatigue (SRF) → academic amotivation (AA). The total effect is defined as the direct effect (cp) plus the serial indirect effect. Confidence intervals are Wald-type 95% CIs based on robust standard errors under WLSMV. Values are rounded to three decimals; *p* values <0.001 are reported as “ <0.001.”

Table 8 compares model fit for the hypothesized serial specification against two competing models, all estimated with WLSMV using ordered categorical indicators (*N* = 518). The hypothesized serial model showed CFI = 0.917, RMSEA = 0.063, and SRMR = 0.048 (χ^2^_scaled = 420.80, df = 139) and served as the reference for ΔCFI. Relative to this baseline, the parallel model demonstrated lower fit (CFI = 0.902, ΔCFI = −0.015; RMSEA = 0.068; SRMR = 0.052; χ^2^_scaled = 471.88, df = 138), and the reverse model likewise showed reduced fit (CFI = 0.906, ΔCFI = −0.011; RMSEA = 0.066; SRMR = 0.053; χ^2^_scaled = 459.86, df = 140). Consistent with these comparisons, the hypothesized serial model exhibited the highest CFI and the lowest RMSEA and SRMR among the three models reported in Table 8.

**Table 8:** Model comparison results for the hypothesized model and competing models (WLSMV; *N* = 518).

Model	χ^2^ (scaled)	df	CFI	ΔCFI	RMSEA	SRMR
1. Hypothesized (serial)	420.80	139	0.917	0.000	0.063	0.048
2. Parallel model	471.88	138	0.902	−0.015	0.068	0.052
3. Reverse model	459.86	140	0.906	−0.011	0.066	0.053

Models were estimated using WLSMV with ordered categorical indicators. ΔCFI is computed relative to the hypothesized serial model (Model 1). Lower RMSEA and SRMR and higher CFI indicate better fit. Values are rounded to three decimals (χ^2^ to two decimals).

## 4 Discussion

### 4.1 Overview of key findings and positioning of the study

This paper investigated the relationship between perceived pressure and uncertainty (PU) and academic amotivation (AA) in a mechanistic cascade shaped by platform-mediated engagement and downstream self-regulatory costs. The hypothesized serial pathway PU → immersive escapism (IE) → self-regulatory fatigue (SRF) → AA was supported in a sample of 518 university students. Both a statistically significant serial indirect effect and a significant remaining direct association between PU and AA were observed. Competing-model comparisons further favored the serial specification over both a parallel and a reverse-order alternative, although causal direction cannot be established given the cross-sectional design (Banker et al., 2026; Li and Hashim, 2025).

### 4.2 Descriptive patterns as context: why digital stress relief is important to mechanism

The descriptive findings provide substantive context for interpreting the downstream pathway: short-video platforms were the most frequently reported tool for digital stress relief, followed by social/text-based communities, long-form video platforms, online games/mobile games, online novels/comics, and AI chat/companion apps. Such distribution is not a usage footnote, but it suggests that the idea of stress relief is actively sought in attention architectures that are designed to run continuously, to be recommended, and to run the continuation process with low friction, which are conditions that likely enhance immersion and make disengagement expensive (Hu and Huang, 2024; Xiong et al., 2024; Xu et al., 2023). In this respect, the descriptive landscape justifies conceptualizing immersive escapism (IE) as more than time online: it reflects a qualitatively distinct engagement state that can channel pressure-related affect regulation into immersive, low-friction coping with downstream self-regulatory costs (Orazi et al., 2023; Soraci et al., 2025).

### 4.3 Measurement evidence and robustness: high structural support, construct tension meaningful

The measurement evidence suggests that the focal constructs were empirically distinguishable while also highlighting meaningful conceptual proximity. Internal consistency was modest across constructs (Cronbach's α = 0.583–0.720; McDonald's ωt = 0.636–0.762), which is understandable given the use of brief ordinal measures, although the relatively weak reliability of academic amotivation warrants caution in interpretation. Importantly, the four-factor CFA solution estimated with WLSMV demonstrated good global fit and supported the adequacy of the measurement model for subsequent structural analyses. Discriminant validity evidence was generally acceptable, although the strongest overlap appeared between IE and SRF (HTMT = 0.856). This pattern suggests that the two constructs are closely connected but not identical: IE reflects a coping-oriented mode of digital immersion, whereas SRF reflects the depletion of regulatory resources that may follow repeated or prolonged reliance on such coping. Future studies should continue refining the boundary between these neighboring constructs and further validate the measurement of academic amotivation in digitally saturated student settings.

### 4.4 Mechanistic explanation of the serial pathway: in what place the model is most effective, and why it is important

The serial mediation findings help explain how pressure-related experiences may be associated with academic motivational erosion through digitally mediated coping. PU was positively associated with IE (β = 0.714, *p* < 0.001), IE was strongly positively associated with SRF (β = 0.905, *p* < 0.001), and SRF was positively associated with AA (β = 0.260, *p* = 0.002). Consistent with the hypothesized chain, the serial indirect effect was statistically significant, while the direct association between PU and AA remained present, indicating partial mediation. From a theoretical perspective, the proposed ordering treats immersive escapism as a coping-oriented response to pressure uncertainty and self-regulatory fatigue as a downstream depletion process that may follow repeated or prolonged reliance on such coping. In this sense, the model does not assume that digital immersion is inherently pathological; rather, it suggests that under conditions of pressure and uncertainty, stress-relief seeking within algorithmically curated environments may become linked to a broader pattern of resource depletion and reduced academic motivation.

### 4.5 Competing models and directionality: support for the hypothesized ordering and indications of possible feedback

The competing-model analysis provided a robustness check on the hypothesized ordering. As shown in Table 8, the hypothesized serial model demonstrated the best overall fit (CFI = 0.917, RMSEA = 0.063, SRMR = 0.048) relative to both the parallel model (CFI = 0.902, RMSEA = 0.068, SRMR = 0.052; ΔCFI = −0.015) and the reverse-order model (CFI = 0.906, RMSEA = 0.066, SRMR = 0.053; ΔCFI = −0.011). These results are more consistent with the proposed ordering than with the competing specifications in the present dataset, but they should not be interpreted as definitive proof of temporal precedence. Alternative orderings and reciprocal feedback processes remain plausible and should be examined in future longitudinal, experimental, or intensive repeated-measures research (Balaskas et al., 2025).

### 4.6 Theoretical contributions: what the research will contribute to the existing screen time discourse

The combined measurement, mediation and model-comparison evidence have three contributions. First, conceptualizing immersive escapism (IE) as a platform-shaped engagement mechanism rather than a proxy for time online clarifies how pressure-related affect regulation may be routed through algorithmically organized attention architectures, extending stress-coping accounts to contemporary digital environments. Second, the results identify a plausible process according to which short-term relief seeking embedded in immersive escapist engagement (IE) is converted into the risk of academic motivational erosion in the long term through self-regulatory fatigue, and coping decisions are related to resource-based limitations of long-term academic activities. Third, the persistence of a substantial direct PU → AA association, as well as a substantial serial indirect effect, is evidence that academic amotivation is influenced by not only a depletion of proximal resources but also more immediate, pressure-related disturbances in meaning and expectancy, which makes it difficult to explain using either individual self-control or generic problematic use labels (de la Fuente et al., 2025; Kong et al., 2025; Qiang et al., 2024).

### 4.7 Practical implications, limitations, and next steps

The findings indicate actionable leverage points along the proposed chain. Upstream interventions that decrease perceived uncertainty and pressure (e.g., clearer academic and career pathways, predictable assessment criteria, and accessible support resources) may curb the initial push toward immersive escapism (IE); midstream literacy and self-management interventions that introduce friction or disruption to recommendation-driven consumption may weaken recommendation-driven, low-friction engagement patterns; and downstream efforts that extend coping repertoires and restore regulatory capacity (e.g., sleep, recovery practices, and structured alternatives to avoidant scrolling) may reduce spillover from self-regulatory fatigue into academic motivation (Balaskas et al., 2025; Sun et al., 2022). In addition, universities may need to move beyond generic “use your phone less” messaging and provide more specific support, such as digital wellbeing education, attention management training, and algorithm-related digital literacy for students who rely on digital environments for stress relief. Platform-side responses may also matter: greater transparency about why specific content is shown, along with pacing or interruption tools, may help reduce the gradual drift from stress relief toward regulatory depletion. Meanwhile, interpretation must be moderated by several limitations: the cross-sectional, self-reported design does not allow unequivocal causal assertions and may be vulnerable to same-source inflation (Podsakoff et al., 2003); the proximity between IE and SRF is relatively high (HTMT = 0.856), raising the possibility of construct overlap; the internal consistency of academic amotivation was relatively modest, suggesting that findings involving this construct should be interpreted with caution; and the sample is limited to one institution, which constrains generalizability. Future studies should therefore re-test the proposed ordering using longitudinal/ESM designs, incorporate behavioral trace data to refine the measurement of immersion, and examine whether the system reflects a one-way pathway or a feedback cycle across peak academic stress periods (Dogan et al., 2022; Huang et al., 2025).

## 5 Conclusion

This research offers converging evidence that pressure uncertainty is associated with academic amotivation through an ordered pathway involving immersive digital coping and self-regulatory fatigue among 518 university students. While the cross-sectional, self-report nature of the study limits causal inference and some neighboring constructs showed substantial proximity, the findings suggest that algorithmically curated digital relief may function as more than a harmless pause from pressure. Under conditions of uncertainty, it may become linked to a broader pattern involving stress, immersion, depletion, and reduced academic motivation. Understanding this pattern can help move digital wellbeing policy beyond blunt screen-time reduction and toward more mechanism-sensitive forms of educational and platform intervention.

## Data Availability

The datasets generated and/or analyzed during the current study are not publicly available due to privacy and ethical restrictions, but are available from the corresponding author on reasonable request.
